# Impact of Exercise on Innate Immunity in Multiple Sclerosis Progression and Symptomatology

**DOI:** 10.3389/fphys.2016.00194

**Published:** 2016-06-02

**Authors:** Alison Barry, Owen Cronin, Aisling M. Ryan, Brian Sweeney, Siew M. Yap, Orna O'Toole, Andrew P. Allen, Gerard Clarke, Ken D. O'Halloran, Eric J. Downer

**Affiliations:** ^1^Department of Physiology, School of Medicine, University College CorkCork, Ireland; ^2^Department of Medicine, Cork University HospitalCork, Ireland; ^3^Department of Neurology, Cork University HospitalCork, Ireland; ^4^Mercy University HospitalCork, Ireland; ^5^Department of Psychiatry and Neurobehavioral Science, APC Microbiome Institute, University College CorkCork, Ireland; ^6^Department of Physiology, School of Medicine, Trinity Biomedical Sciences Institute, Trinity College Dublin, University of DublinDublin, Ireland

**Keywords:** Multiple Sclerosis, exercise, neuroinflammation, innate immunity, TLRs, cytokines

## Abstract

Multiple Sclerosis (MS), an idiopathic progressive immune-mediated neurological disorder of the central nervous system (CNS), is characterized by recurrent episodes of inflammatory demyelination and consequent axonal deterioration. It accounts for functional deterioration and lasting disability among young adults. A body of literature demonstrates that physical activity counteracts fatigue and depression and may improve overall quality of life in MS patients. Furthermore, much data indicates that exercise ameliorates chronic neuroinflammation and its related pathologies by tipping cytokine profiles toward an anti-inflammatory signature. Recent data has focused on the direct impact of exercise training on the innate immune system by targeting toll-like receptors (TLRs), signaling pattern recognition receptors that govern the innate immune response, shedding light on the physiological role of TLRs in health and disease. Indeed, TLRs continue to emerge as players in the neuroinflammatory processes underpinning MS. This review will highlight evidence that physical activity and exercise are potential immunomodulatory therapies, targeting innate signaling mechanism(s) to modulate MS symptom development and progression.

## Multiple sclerosis

Multiple Sclerosis (MS) is an immune-mediated demyelinating disorder of the central nervous system (CNS; Calabresi, 2004; Goldenberg, 2012), which is the most common cause of acquired non-traumatic neurological disability among young adults (Compston and Coles, 2002; Zipp and Aktas, 2006), predominantly affecting those between the age of 20–40 years (Comabella and Khoury, 2012). MS is regarded as an autoimmune disease since inflammatory lesions associated with the disease are well-characterized by blood brain barrier (BBB) leakage and massive lymphocytic infiltration, principally the participation of the CD4^+^ T cells (Brück, 2005; Comabella and Khoury, 2012). Both white and gray matter are affected by neurodegenerative and inflammatory mechanisms (Kutzelnigg et al., 2005; Crespy et al., 2011). This neurological damage can result in the characteristic spectrum of presenting symptoms including paraesthesia, numbness, muscle weakness, gait imbalance, spasticity, cerebellar ataxia, visual impairment, dizziness, urinary dysfunction, fatigue, depression and cognitive abnormalities (Andersson et al., 1999; Calabresi, 2004). These symptoms interfere with activities in everyday life and negatively impact quality of life. Additionally, MS is a costly disease, such that in 2010 the costs (direct and indirect) associated with MS in Europe alone reached a staggering €14.6 billion (Gustavsson et al., 2011). It is clear that MS is economically, medically and societally burdensome.

### Etiology and pathogenesis

**Figure 1:**
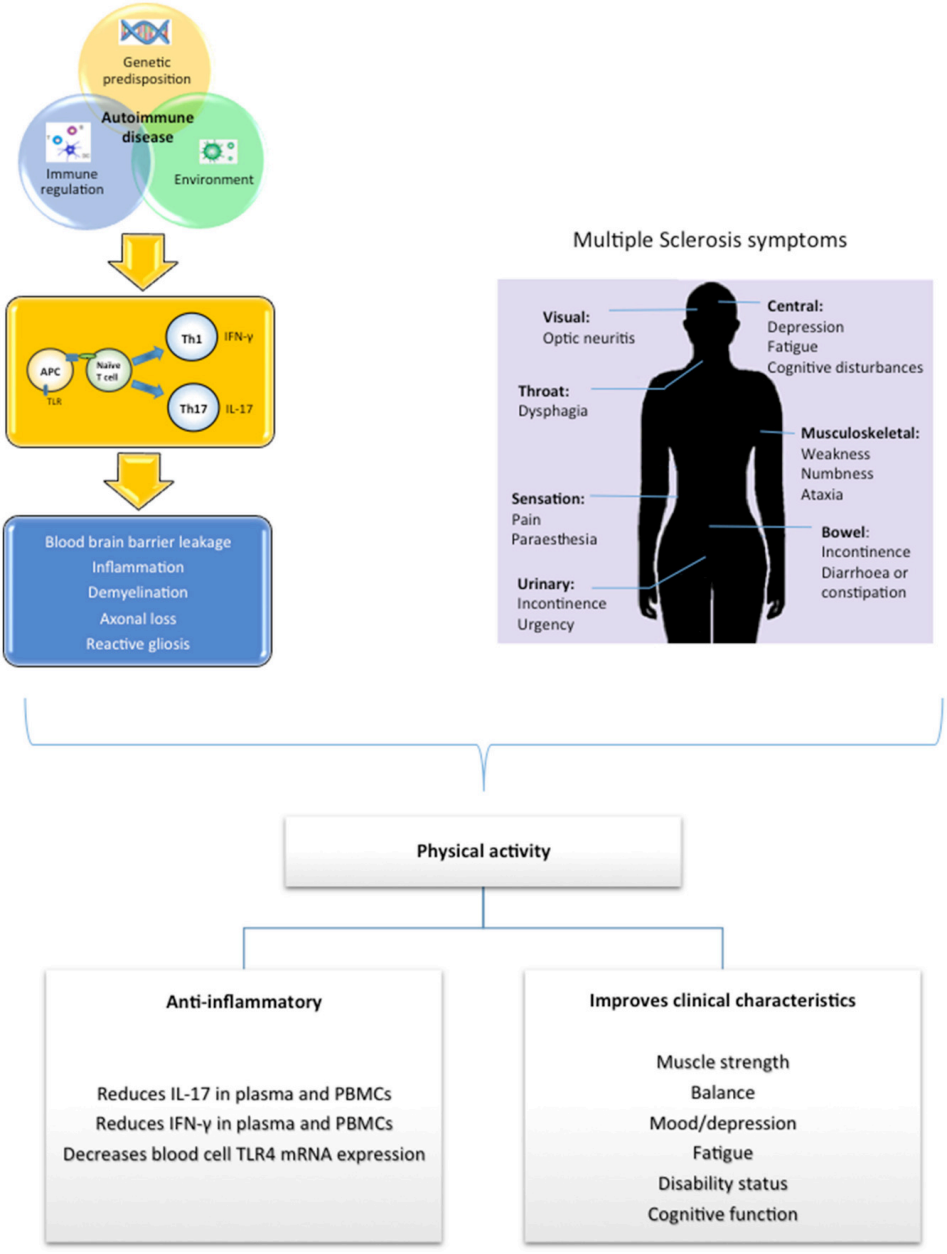
**Susceptibility to MS is believed to be caused by the complex interaction between genetic, environmental and immunological factors which triggers an immune attack and the initiation of MS**. Physical activity has been shown to target inflammatory and immune genes associated with MS neuropathology, exerting an anti-inflammtory effect. Additionally many common presenting symptoms associated with MS have been shown to be improved in response to physical activity.

The pathogenesis of MS is highly complex with the identity of a single unifying cause underlying its etiology remaining elusive; however it is believed that an intricate interplay between immunological factors, genetic factors and environmental influences determines susceptibility to the disease (Figure 1; Comabella and Khoury, 2012). MS can be characterized by heterogeneous histopathological modifications associated with the presence of multi-focal regions of demyelination, inflammation, axonal loss and reactive gliosis distributed throughout the CNS (Brück, 2005; Dutta and Trapp, 2011). While initial CNS damage in MS is linked with immune-mediated destruction of myelin and oligodendrocytes, it is suggested that later progressive axonal degeneration is responsible for neurological disability in MS (Dutta and Trapp, 2011).

Historically, MS has been regarded as an adaptive immune response through the activation of autoreactive myelin specific T-cells, for example activation of autoreactive CD4^+^ T cells and their differentiation into T-helper 1 (Th1) cells is of particular importance in MS (Fletcher et al., 2010; Rostami and Ciric, 2013). CD4^+^ T cells have shown specificity for myelin proteins, such as myelin basic protein (MBP), myelin associated glycoprotein (MAG), myelin oligodendrocyte glycoprotein (MOG), and proteolipid protein (PLP), leading to tissue damage and contributing to lesion formation (Sospedra and Martin, 2005). However, involvement of innate immunity in both initiation and progression of the disease has become increasingly recognized (Gandhi et al., 2010; outlined below). The innate immune system is highly conserved and it is the first line of defense against invading pathogens by orchestrating cells of the immune system including neutrophils, macrophages and dendritic cells (DC) (Akira et al., 2003). These cells play a crucial role in discriminating between pathogens and self through the action of toll-like receptors (TLRs) and by initiating tailored immune responses to eliminate the invading pathogen. Much evidence indicates that dysregulation or over-activation of the innate immune response is associated with neuro-inflammatory processes which has been linked with numerous neurodegenerative disorders, including MS (De Faria et al., 2012).

### Current therapeutic strategies in MS

Currently available immunomodulatory therapies for MS include injectable medications such as interferon (IFN)-β (betaseron®, extavia®, avonex®, rebif®) and glatiramer acetate (copaxone), oral medications such as fingolimod (gilenya®) dimethyl fumarate (tecfidera®) and teriflunomide (aubagio®), and infused medications such as natalizumab (tysabri®), alemtuzumab (lemtrada™), and mitoxantrone (novantrone®; Table 1). Each medication is partially effective in reducing relapse rate and slowing the progression of the disease, however, they are not without side effects and complications.

**Table 1:** **Summary of immunomodulatory therapies for MS**.

**Generic Name**	**Trade Name(s)**	**Route of administration**	**Proposed mechanism's of action**
Beta interferon 1a	Avonex®, Rebif®	Intramuscular/Subcutaneous	Downregulates T cell activity and inflammatory cytokines
Beta interferon 1b	Betaseron®	Subcutaneous	Downregulates T cell activity and inflammatory cytokines
Glatiramer acetate	Copaxane	Subcutaneous	Inhibits MBP reactive T cell activation and increases Th2 cell population
Fingolimod	Gilenya®	Oral	Prevents lymphocyte egress from lymph nodes
Dimethlyfumarate	Tecfidera®	Oral	Inhibits expression of cytokine and inflammatory molecules
Alemtuzumab	Lemtrada^™^	Infused	Depletes circulating lymphocytes
Natalizumab	Tysabri®	Infused	Blocks lymphocyte migration into the CNS
Teriflunomide	Aubagio®	Oral	Inhibits DHODH & reduces lymphocyte proliferation
Mitoxantrone	Novantrone®	Infused	Targets T/B cell activity and macrophage proliferation

*DHODH, dihydro-orotate dehydrogenase; MBP, myelin basic protein*.

### Injectable medications

Avonex® and rebif® (both IFN-β-1a), and betaseron® and extavia® (IFN-β-1b), exert effects on the BBB and the activity of lymphocytes (Mendes and Sá, 2011), although their precise mechanisms of therapeutic action remain unclear. Furthermore IFN-β drugs reduce relapse rate and the development of new lesions as shown by MRI analysis (Paty and Li, 1993). Copaxone is a synthetic mixture of four amino acids found in MBP which binds major histocompatibility complex (MHC) molecules and competes with endogenous myelin antigens for T cell recognition, stimulating an anti-inflammatory signature with an increase in Th2 cell migration to the brain (Teitelbaum et al., 1999). Furthermore, data indicates that copaxone may also exert neuroprotective effects in the CNS by increasing the expression of brain-derived neurotrophic factor (BDNF; Ziemssen et al., 2002).

### Oral medications

Gilenya® modulates sphingosine-1 phosphate receptor activity resulting in prevention of lymphocyte egress from lymph nodes (Matloubian et al., 2004), thus reducing infiltration into the CNS and immune-mediated damage. Additionally, research evidence indicates that gilenya® targets oligodendrocyte progenitor cells which are directly responsible for remyelination (Miron et al., 2008). Overall gilenya® treatment reduces relapse rate, disability progression and T2 lesion volume (Kappos et al., 2010). Tecfidera® may exert anti-inflammatory effects by inhibiting the expression of cytokines and adhesion molecules, in addition to exerting neuroprotective effects through activation of the nuclear-factor-E2-related factor-2 (Nrf2) transcription pathway which protects neurons from oxidative stress, prevents BBB breakdown and maintains myelin integrity (Kappos et al., 2008). Tecfidera® treatment is also associated with a reduced number of T2- and T1-hypertensive lesions and reduced relapse rate (Kappos et al., 2008). Aubagio® is the active metabolite of leflunomide which was initially approved for the treatment of rheumatoid arthritis (Rozman, 1998). Therapeutically, aubagio® acts as an immunosuppressant, by inhibiting the mitochondrial enzyme dihydro-orotate dehydrogenase (DHODH) which is required for the *de novo* synthesis of pyrimidine and thus reduces DNA synthesis, overall exerting a cytostatic effect on B and T cell proliferation (Cherwinski et al., 1995; Greene et al., 1995). O'Connor et al. (2011) demonstrated that aubagio® reduces relapse rate, disability progression and disease activity as shown by MRI (O'Connor et al., 2011).

### Infused medications

Tysabri® is a monoclonal antibody that therapeutically modulates immune responses in MS. Tysabri® has specificity for the α4-integrin receptor subunit on activated T cells, antagonizes cell adhesion to vascular endothelium at the BBB, and hence inhibits immune cell infiltration into the CNS (Yednock et al., 1992), preventing the destruction of myelin and the impairment of nerve conduction. Tysabri® has been shown to reduce relapse rate, disability progression rate and the number of T2-hyperintense lesions (Havrdova et al., 2009). Lemtrada^™^ is a monoclonal antibody infused for 5 consecutive days every 12 months which results in a rapid and prolonged depletion of circulating lymphocytes, followed by a homeostatic repopulation of regulatory T cells and memory B and T cells, thus improving disability and suppressing clinical exacerbations (Coles, 2013). Finally, novantrone® is an immunosuppressant that targets T/B cell and macrophage proliferation, in addition to altering T and B cell activity, augmenting T cell suppressor function and inhibiting B cell function and the production of antibodies (Fox, 2004). Novantrone® also reduces disability progression and relapse rate (Hartung et al., 2002).

## Innate immune system

### General overview

**Figure 2:**
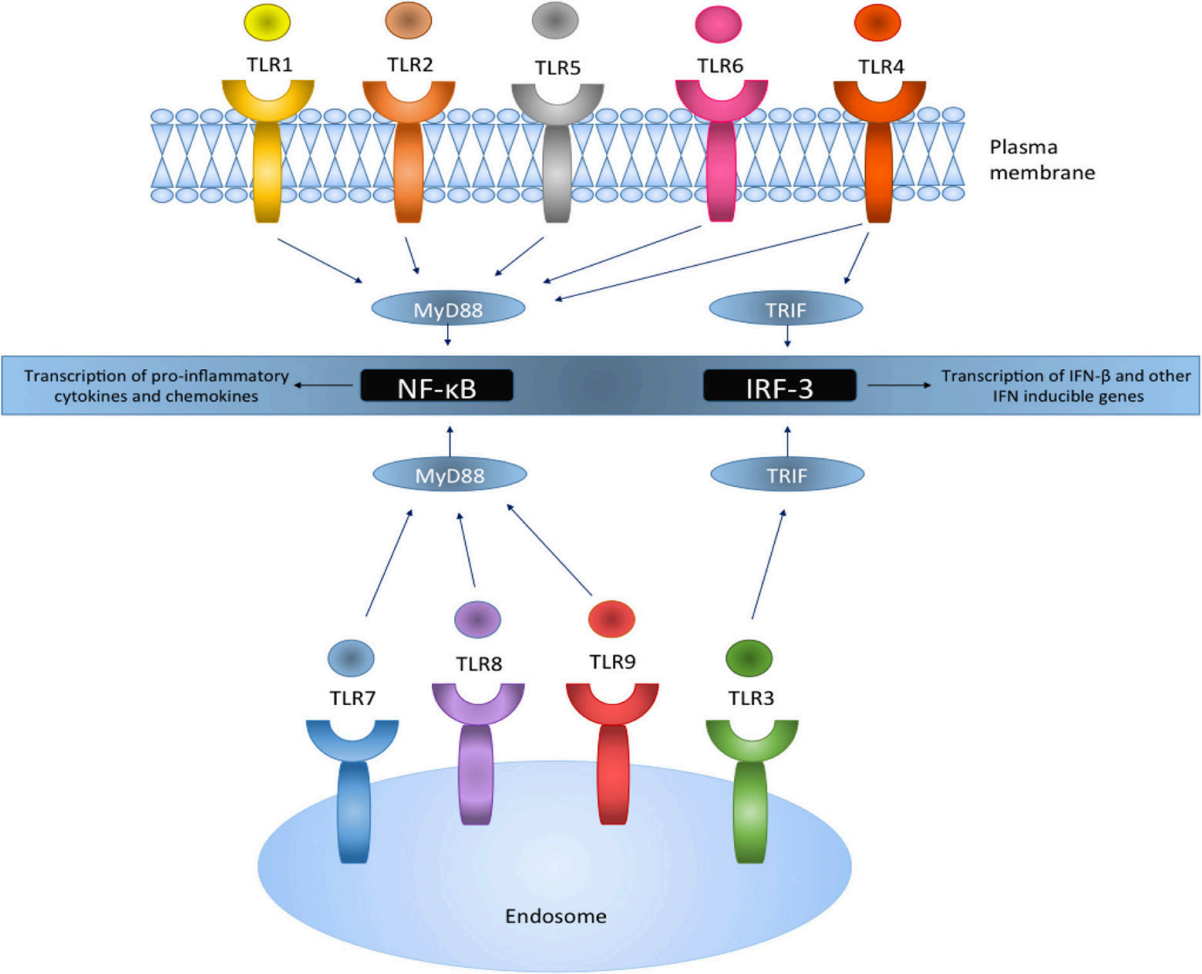
**TLRs reside on the plasma membrane or intracellularly on endosomes and act via the MyD88 or the TRIF pathway leading to the activation of downstream signaling pathways**. TLR activation results in the translocation and transcriptional activation of transcription factors NF-κB and IRF-3, and the production of pro-inflammatory genes and IFN inducible genes, respectively.

Playing an essential role in immunity, the innate immune system is recognized as the first line of host defense, providing immediate protection against pathogenic infectious agents through initiating complex interactions between the pathogen and the immune mechanisms of the host (Kumar et al., 2011). Macrophages and other cells in the innate immune system express pathogen recognition receptors (PRRs), which recognize pathogen-associated molecular patterns (PAMPs) expressed by pathogens (Kumar et al., 2011). This interaction stimulates the release of a multitude of mediators responsible for an inflammatory response including cytokines, chemokines and type 1 IFNs (Figure 2). Thus, the innate immune system provides early recognition and immediate protection by facilitating the eradication of the pathogen, and furthermore regulates the initiation of the adaptive immune response (Mogensen, 2009).

### TLR signaling

TLRs are a family of innate immune system receptors which either span the cell membrane or are expressed intracellularly on endosomes in both nonimmune and immune cells (Figure 2), most notably macrophages and DCs (Hernández-Pedro et al., 2013). TLRs are members of the PRR family, which recognize distinct exogenous conserved pathogenic motifs, PAMPS, and also endogenous damage-associated molecular patterns (DAMPS) from necrotic or dying cells (Mills, 2011). TLR activation results in anti-microbial responses and the production of pro-inflammatory cytokines, chemokines and IFNs. To date, 10 functional human TLRs have been identified, while 13 murine TLRs characterized (Capelluto, 2012), each of which detects different microbial components (Table 2).

**Table 2:** **Examples of pathogen-derived, endogenous and synthetic ligands for TLRs**.

**Receptor**	**Expression**	**Pathogen-derived ligands**	**Endogenous ligands**	**Synthetic ligand**
TLR1	Extracellular	Bacteria: peptidoglycan		Pam_3_CSK_4_
TLR2	Extracellular	Bacteria: lipoproteins	Heat shock	Pam_3_CSK_4,_ MALP-2
		Fungi: zymosan	HMGB1,veriscan	
TLR3	Intracellular	Virus: dsRNA	mRNA	Poly(I:C)
TLR4	Extracellular	Bacteria: LPS	Saturated fatty acids	Lipid derivatives
		Virus: fusion protein	Amyloid-β	
		Fungi: mannan		
TLR5	Extracellular	Bacteria: flagellin		
TLR6	Extracellular	Bacteria: lipoteichoic acid	Veriscan	
TLR7	Intracellular	Virus: ssRNA	Self RNA	Imidazoquinoline, Bropirimine
TLR8	Intracellular	Virus: ssRNA	Self RNA	Imidazoquinoline,
TLR9	Intracellular	Bacteria: CpG- DNA	Self RNA	CpG-ODN
		Virus: CpG- DNA		
TLR10	Extracellular	Virus: H5N1, H1N1		

*HMGB1, High mobility group box 1 protein; LPS, lipopolyscaccharide*.

TLRs are type 1 transmembrane proteins and are comprised of a cytoplasmic Toll/IL-1R (TIR) domain and an extracellular leucine-rich repeat (LRR) domain (Singh and Naik, 2005). After stimulation by TLR ligands, all TLRs recruit specific adaptor proteins via a conserved TIR domain and a death domain, which activate downstream signaling cascades ultimately leading to the activation of transcription factors such as nuclear factor-κB (NF-κB) and IFN-regulatory factors (IRFs; O'Neill and Bowie, 2007; Figure 2). This promotes the transcriptional activation of genes responsible for encoding pro-inflammatory chemokines and cytokines which subsequently stimulate innate immune responses and prime antigen specific adaptive immune responses (Jarrossay et al., 2001; Singh and Naik, 2005; Hernández-Pedro et al., 2013).

All TLR (apart from TLR3) signaling involves the recruitment of a key adaptor protein, myeloid differentiation factor 88 (MyD88). Additional adaptor proteins, including MyD88-adapter-like (MAL) and TRIF-related adaptor molecule (TRAM) both act as bridging adaptors, with MAL facilitating the interaction of MyD88 with TLR4 to promote NF-κB activation, whereas TRAM recruits the adaptor protein Toll/IL-1R domain-containing adaptor inducing IFN (TRIF) which enables IRF-3 activation (O'Neill and Bowie, 2007). Hence, both the MyD88-dependent and TRIF-dependent (MyD88-independent pathway) activation pathways are facilitated by these adaptor proteins. TLR4 can use both the MyD88 and TRIF pathway to initiate the production of pro-inflammatory cytokines and IFN-stimulated genes (Selvarajoo et al., 2008), whereas TLR3 utilizes the TRIF pathway.

TLR4 is localized on the cell surface of immune cells and cells of the CNS, and is activated primarily to bacterial PAMPS such as gram negative bacteria lipopolysaccharide (LPS). Recognition also requires the accessory molecule MD2 (Kim et al., 2007). Once TLR ligands bind to the receptor, MyD88 and other associated adaptor proteins are recruited. Indeed, MyD88 further recruits and forms a complex with a member of the IL-1R-associated kinase (IRAK) family through their death domains (Akira et al., 2003) and toll-interacting protein (Tollip; Capelluto, 2012). This enables IRAK autophosphorylation and its dissociation from the complex whereby it interacts with downstream adaptor tumor necrosis factor alpha (TNF-α) receptor-associated factor 6 (TRAF-6; Akira et al., 2003). TRAF-6 then activates transforming growth factor-β (TGF-β)-activating kinase (TAK1) which sequentially activates downstream IκB kinases (IKK). Members of the inhibitory IκB family which usually sequester NF-κB in the cytosol, are then directly phosphorylated by IKKs, enabling NF-κB translocation into nucleus to induce target gene expression (Moynagh, 2005). NF-κB inducible genes are responsible for the encoding pro-inflammatory cytokines and chemokines including interleukin (IL)-1, IL-6, IL-8, and TNF-α (Mogensen, 2009).

TLR3 resides in endosomal compartments of immune (particularly in DC and B cells) and CNS cells, and recognizes distinct viral double stranded nucleic acid RNA (Singh and Naik, 2005). TLR3 activation results in the recruitment of the TRIF adaptor protein, initiating a signaling pathway through TRAF3 (Häcker et al., 2006), TANK-binding kinase 1 (TBK1) and IKK; this signaling complex then mediates phosphorylation of IRF-3 (Fitzgerald et al., 2003), enabling its translocation to the nucleus where it binds target DNA sequences, facilitating transcriptional activation of genes responsible for encoding type I IFNs (Akira et al., 2003). The TRIF pathway can lead to activation of both NF-κB and IRF-3 (Akira and Takeda, 2004), and indeed TBK1 and IKK are also implicated in the activation of NF-κB through the TRIF-dependent pathway (Fitzgerald et al., 2003). TLR4 can also signal via the MyD88-independent pathway through the bridging adaptor TRAM, promoting IRF-3 activation and IFN-β induction.

### Role of innate immunity in MS

Infections have been thought to increase susceptibility to autoimmune diseases. For example, many observations implicate Epstein–Barr virus (EBV) in the pathogenesis of MS (Lünemann et al., 2007). Furthermore, viral infections have been shown to correlate with MS attacks and exacerbation of symptoms, presumably through the modification of the immune system in response to exogenous events (Panitch, 1994). The most common mechanism by which pathogens are suggested to trigger or exacerbate autoimmunity is through molecular mimicry; whereby pathogens possess antigens of similar sequence and structure to host T or B cell epitopes. Thus, upon infection this may result in the initiation of a self-specific immune response (Cusick et al., 2012), leading to a cascade of detrimental effects causing prolonged pro-inflammatory responses and subsequent tissue and organ degeneration.

MS is driven by pro-inflammatory chemokines and cytokines (Sørensen et al., 1999). Elevated levels of Th1 and Th17 cell cytokines such as IL-1, IL-6, IL-12, IL-17, IFN-γ, and TNF-α in particular, infiltrate the CNS and play a crucial role in MS pathogenesis. Recovery and remission periods in MS are also associated with altered CNS expression profiles of the anti-inflammatory Th2 cell type cytokines, including IL-10 (Ozenci et al., 2002; Florindo, 2014). Indeed, the use of cytokine signature patterns may help to stratify drug treated and drug naïve patient groups (O'Connell et al., 2014). Although it is unknown what triggers the initial immune response in MS, the innate immune system is suggested to play a role as both a promoter and mediator of the disease (Gandhi et al., 2010).

Infiltrating and resident cells within the CNS both express TLRs and an increase in TLR expression has been observed in autoimmune diseases such as MS (Miranda-Hernandez and Baxter, 2013), even in the absence of a microbial environment. Microglia cultured from human cerebral tissue express mRNA encoding TLRs 1-9, while astrocytes primarily express robust levels of TLR3, as shown by quantitative real-time PCR. Ligation of TLR3 and TLR4, by poly(I:C) and LPS respectively, leads to microglial activation and consequently stimulates secretion of cytokines IL-12, TNFα, IL-6, IL-10 and chemokine CXCL-10 (a chemoattractant for pro-inflammatory T cells), which are strongly associated with MS pathogenesis (Jack et al., 2005). Interestingly, increased *in vivo* expression of TLR3 and TLR4 have been identified in MS brain and spinal cord sections in comparison to controls, as shown by immunohistochemical analysis (Bsibsi et al., 2002). Additionally, TLR2 expression in oligodendrocytes is upregulated in MS lesions, repressing episodes of remyelination (Sloane et al., 2010).

Using experimental autoimmune encephalomyelitis (EAE), the murine model of MS, specific roles of TLRs have been indicated in EAE. Indeed, stimulation of TLR3 with poly(I:C) has been demonstrated to suppress the development of a murine model of relapsing EAE, presumably through enhanced levels of IFN-β and the chemokine, CCL2 (Touil et al., 2006). Thus, it is suggested that TLR3 signaling through the MyD88-independent pathway suppresses, or does not support, the development of EAE. This is significant given that IFN-β is a first line treatment in RRMS patients. Furthermore, Guo et al. (2008) demonstrated that type 1 IFN receptor knockout mice and TRIF knockout mice developed more severe EAE than wild type mice, manifested by enhanced levels of IL-17 production (Guo et al., 2008). This illustrates the importance of TRIF-dependent IFN-β production and downstream signaling in suppressing the development of Th17 cells and autoimmune inflammation.

Alternatively, TLR signaling through the MyD88-dependent pathway is believed to play a part in the development of EAE. Indeed, Marta et al. (2008) demonstrated that MyD88 deficient mice were completely resistant to EAE development and subsequently these mice exhibited reduced splenic myeloid dendritic cell (mDC) IL-6 and IL-23 expression, and Th17 responses were absent (Marta et al., 2008). Further support for the involvement of MyD88 as a key modulator of autoimmunity was provided by Prinz et al. (2006) who showed that MyD88 knockout mice did not respond to immunization and were indeed resistant to active EAE (Prinz et al., 2006).

Pertussis toxin (PT) administered at the time of immunization is suggested to regulate P-selectin expression and enhance leukocyte/endothelial cell interactions, facilitating T cell infiltration into the CNS by increasing BBB permeability. PT induces TLR4 signaling and consequently controls leukocyte recruitment in wild type mice. These effects were not detected in TLR4 knockout mice and they were less susceptible to PT-induced EAE in comparison to wild type mice (Kerfoot et al., 2004). These findings suggest that TLR4 signaling participates in the initiation of EAE. In contrast, Marta et al. (2008) demonstrated that TLR4 knockout mice presented enhanced levels of mDC (IL-6 and IL-23) and an increase in the Th17 population, overall presenting more severe clinical symptoms than the wild type animals (Marta et al., 2008). Interestingly, germ-free mice, characterized by impaired innate immune responses and reduced TLR4 expression (Wang et al., 2010), display significantly attenuated EAE (Lee et al., 2011), overall indicating the complex role of TLR4 in EAE pathogenesis.

Differential TLR responses of immune cells isolated from MS patients also suggest the complex role of TLR signaling in MS pathogenesis. Indeed, Crowley et al. (2015) recently demonstrated that peripheral blood mononuclear cells (PBMCs) isolated from MS patients are hypersensitive to TLR4 stimulation, promoting a pro-inflammatory signature (Crowley et al., 2015). Downer et al. (2011) also demonstrated that PBMCs from MS patients are refractory to poly(I:C) treatment, in terms of IFN-β expression, thus indicating that blood cells from MS patients may show TLR3 tolerance (Downer et al., 2011).

## Impact of exercise on the innate immune system

Exercise activates an array of immunological and hormonal responses, and much evidence indicates that exercise training can ameliorate chronic neuroinflammation and its related pathologies by targeting pro- and anti-inflammatory cytokines (Florindo, 2014). Indeed, in chronic illness, exercise can skew cytokine profiles toward an anti-inflammatory signature, which contributes to the health benefits of exercise and protects against chronic diseases associated with low-grade inflammation (Petersen and Pedersen, 2005). In addition, new data now indicates that exercise training directly impacts the innate immune system by targeting TLR signaling, which sheds light on the physiological regulation of TLR expression and function in humans in health and disease.

### Acute strenuous exercise (one exercise session) in healthy human subjects

Acute exercise can profoundly affect immune cell profiles, particularly during and immediately after exercise. Indeed, marathon running enhances the circulating DC population, while decreasing the number of plasmacytoid DC, in healthy elite and non-elite runners, suggesting that immunomodulatory mechanisms are central in the response to acute excessive exercise (Nickel et al., 2011). In the same study Nickel et al. (2011) demonstrate that the levels of IL-6, IL-10, TNF-α, and C-reactive protein (CRP) are increased in serum samples post-marathon (Nickel et al., 2011). Similarly, circulating levels of neutrophils, plasma cytokines (IL-6 and IL-10) and neutrophil TLR4 and IRAK3 expression are enhanced in healthy-endurance trained individuals undertaking a single exercise trial (cycling for 1 h at 105% power output followed immediately by running for 1 h at ~10-km time trial pace; Neubauer et al., 2013). In support of this, Booth et al. (2010) demonstrate that cycling (60 km time trial at fastest pace) increases the total cell numbers of neutrophils, lymphocytes and monocytes, while also increasing the expression of TLR2 and TLR4 in monocytes isolated from elite cyclists immediately after exercise (Booth et al., 2010). Their findings also indicate that the expression of the MHC class II receptor HLA.DR, is reduced on monocytes following the time trial (Booth et al., 2010). The stimulatory effect of acute exercise on plasma IL-6 has also been identified immediately following a shorter bout of ergometry training (60% of VO_2_max for 30 min) in healthy volunteers (and RRMS patients; Castellano et al., 2008). Interestingly, Sureda et al. (2014) have recently demonstrated that acute exercise associated with a single bout of scuba diving (50 m depth for 35 min), enhanced mRNA levels of a panel of inflammatory genes in neutrophils, including *NF*-κ*B, TLR4, COX2, IL-6, IL-8, IL-10, IL-1*, and *iNOS*, demonstrating that the acute exercise associated with scuba diving enhances the inflammatory response in neutrophils (Sureda et al., 2014). Given that strenuous aerobic cycling (two repeated bouts of 50% of VO_2_max for 60 min) in healthy volunteers enhances plasma non-esterified fatty acids (NEFAs; Stich et al., 2000), alongside evidence that circulating levels of LPS are enhanced following completion of an ultra-distance triathlon in healthy triathletes (Bosenberg et al., 1988), a single bout of exercise may upregulate innate immune signaling mechanism via TLR stimulation in human blood.

It is important to note that several studies indicate that acute exercise reduces innate immune receptor expression, and may account for post-exercise immuno-depression. Indeed, marathon running reduces the expression of TLR7 on PBMCs (Nickel et al., 2011) and moderate intensity exercise (1.5 h of cycling exercise at ~65% VO_2_max) in the heat (34°C in 30% relative humidity) reduces the expression of TLRs (1, 2, and 4), CD86 and MHCII in monocytes in healthy volunteers (Lancaster et al., 2005). In parallel, the stimulatory effect of TLR agonists zymosan (for TLR2/6), LPS (for TLR4), or poly(I:C) (for TLR3) on markers of monocyte activation (CD80, CD86, MHCII, and IL-6) is ameliorated following exercise compared with at rest (Lancaster et al., 2005). In support of this, prolonged cycling (1.5 h at 75% VO_2peak_) in healthy subjects increases to total number of circulating monocytes, while reducing monocyte TLR4 protein expression, which may in part be responsible for post-exercise immuno-depression (Oliveira and Gleeson, 2010).

### Chronic exercise (repeated exercise sessions) in healthy human subjects

A body of literature in human trials indicates the impact of chronic exercise on cytokine and inflammatory signaling networks may have a dose-dependent relationship with exercise intensity. Indeed, the immunomodulatory effect of repeated bouts of exercise is well characterized. Resistance training (72 exercise sessions over 6 months) in healthy elderly women reduces TLR4 expression on monocytes (McFarlin et al., 2004), while resistance training (16 resistance training sessions over 8 weeks) in healthy elderly individuals has been shown to be anti-inflammatory in PBMCs, reducing cellular protein expression of TLR2, TLR4, MyD88, TRIF, NF-κB, IKKi/IKKε, and phospho-IRF-3/7 (Rodriguez-Miguelez et al., 2014). In addition, Shimizu et al., 2011) have demonstrated that resistance training (leg extension, leg press, hip abduction, and hip adduction) twice a week for 12 weeks in elderly control subjects increases the number of CD28-expressing T cells and CD80-expressing monocytes, indicating that training may upregulate monocyte and T-cell-mediated immunity in elderly individuals (Shimizu et al., 2011). Finally, Lambert et al. (2008) have shown that a combination of resistance and aerobic training (90 min/day, 3 days/week for 12 weeks) in a cohort of obese elderly individuals reduces the gene expression of *IL-6* (and *TNF*-α and *TLR4*) in skeletal muscle (vastus lateralis) (Lambert et al., 2008). Overall, it is evident that both acute and repeated chronic bouts of exercise training directly impacts the expression profile of TLRs in blood cells, cytokine signature patterns and the function of the innate immune system in healthy individuals.

### Acute and chronic exercise in animal studies

It is well known that exercise is anti-inflammatory and promotes neuroregeneration, plasticity and memory in rodents (Bechara et al., 2013). Indeed, resistance training (predominantly composed of concentric forces) in rats (exercised for 12 weeks; two times per day; two times per week), decreased *TNF*-α mRNA expression in the plantar muscle (Zanchi et al., 2010). In contrast, exhaustive exercise in rats (treadmill running at 70% VO_2_max for 50 min followed by an elevated rate that increased at 1 m/min until exhaustion) increases both TNF-α and IL-10 (gene and protein expression) in both extensor digitorum longus and soleus muscles (Rosa Neto et al., 2009). This group have also demonstrated that exhaustive exercise in rats enhances MyD88, TRAF-6 and IκBα expression in adipose tissue within 6 h post-exercise (Stich et al., 2000). In contrast, data elsewhere indicates that exercise training downregulates pro-inflammatory cytokine gene expression in adipose tissue. Indeed, exercise (65-70% VO_2_max) on motorized treadmills (40 min/day, 5 days/wk, 6 or 12 wk) reduces *TNF*-α expression in adipose tissue of obese mice (Vieira et al., 2009). In support of this, Kawanishi et al. (2010) indicate that exercise (12–20 m/min for 60 min/day for 16 weeks) significantly inhibits *ICAM-1* gene expression in adipose tissue of obese mice, and suggest that exercise may promote the phenotypic switching from M1 macrophage to M2 macrophage in obese adipose tissue (Kawanishi et al., 2010). Indeed, running mice on a treadmill to the point of exhaustion significantly lowers plasma levels of both TNF-α and IFN-α concentrations, indicating that exhaustive exercise can result in immune-depression (Yano et al., 2010). In support of this, treadmill running in rats (12 m/min for 30 min/day, 5 days a week for 2 weeks) blunted the enhanced expression of TLR2, TLR4, NF-κB, and MyD88 in rat cortex after middle cerebral artery occlusion-reperfusion (Ma et al., 2013).

## Exercise and MS

Overall, an imbalance between pro- and anti-inflammatory cytokines exists in MS, exhibiting a shift toward a pro-inflammatory cytokine profile. This makes pro-inflammatory cytokines a good therapeutic target. Numerous sources have indicated that regular exercise can reverse chronic inflammation, with evidence indicating that physical activity decreases pro-inflammatory cytokines as well as promoting an increase in anti-inflammatory cytokines. Indeed in MS, evidence suggests that regular exercise can induce anti-inflammatory effects and may be beneficial in the modulation of MS progression (Figure 1).

### Exercise and the immune system in MS—animal studies

Using EAE, the murine model of MS, several studies have indicated that exercise can combat the clinical development of the disease (Rossi et al., 2009). Indeed, Bernardes et al. (2013) indicate that swimming exercise in mice (30 min/day, 5 days/week for 6 weeks) reduces the severity of EAE while decreasing the protein expression of IL-1, IL-6, TNF-α, and IL-10 in the brain post-EAE induction (Bernardes et al., 2013). In a recent study by a same group, forced swimming exercise in mice (30 min/day, 5 days/week for 6 weeks) was shown to attenuate the number of B and T cells infiltrating the spinal cord in EAE mice (Bernardes et al., 2016). Voluntary wheel running in mice (1 h daily access for 3 days prior to EAE, and each day 1 post EAE induction) delays the onset of EAE and reduces the number of CD45-positive leukocytes and CD^+^ T cells infiltrating the spine (Benson et al., 2015). This exercise protocol also reduced the number of Iba-1-positive microglia in the spinal cord at the onset of EAE (Benson et al., 2015). Interestingly, forced treadmill training in rats (15-30 m/min for 60 or 90 min/day over 10 consecutive days) failed to impact clinical disability development in EAE groups, and furthermore failed to regulate the EAE-induction of total brain TNF-α (Patel and White, 2013). These findings indicate that exercise has the proclivity to target disease development in EAE by targeting glia, immune cells and cytokine signatures, and that these effects are dependent on the type of exercise training.

### Exercise and the immune system in MS—human studies

In MS patients, Golzari et al. (2010) have demonstrated that combined exercise involving endurance and resistance training for 24 sessions during 8 weeks, significantly reduced IL-17 and IFN-γ production in plasma and PBMCs in female MS patients [Expanded Disability Status Scale (EDSS) score of 0–4; Golzari et al., 2010]. IL-17 is one of the most important pro-inflammatory cytokines in MS progression (Stromnes et al., 2008), and IL-17 (mRNA and protein) has been demonstrated in perivascular lymphocytes, astrocytes and in active regions of MS lesions (Tzartos et al., 2008). In addition IL-17 has been identified to play a crucial role in the development of EAE by disrupting the BBB (Huppert et al., 2010). Therefore significant reduction in IL-17 production in response to physical activity would demonstrate a beneficial anti-inflammatory effect in MS patients. These findings are supported elsewhere. Castellano et al. (2008) showed that following 30 min of aerobic exercise at 60% VO_2peak_, IFN-γ plasma concentration was significantly reduced from baseline 3 h post-exercise in RRMS patients (EDSS of 0–5.5; medications included copaxone, rebif® and avonex®; Castellano and White, 2008). However, chronic exercise over 8 weeks tended to increase IFN-γ plasma concentration in MS patients (Castellano and White, 2008). This highlights the differential effects of exercise on plasma IFN-γ. IFN-γ is a potent pro-inflammatory cytokine and can stimulate the production of the cytokine IL-12 which further promotes Th1 type immune responses (Ozenci et al., 2000). Similarly, White et al. (2012) assessed several immune markers in PBMCs from RRMS (immunomodulatory treatment included IFN-β and copaxone) patients undertaking a single session of combined arm and leg exercise (70% of age-predicted maximal heart rate using a Schwinn Air-Dyne ergometer; White et al., 2012). Importantly their findings indicated that blood cells from MS patients had a greater post-exercise (0.5 and 8 h) decrease in the expression on the *TLR4* mRNA, while *IL-6* and *IL-10* mRNA were decreased immediately after exercise (0.5 h; White et al., 2012). Overall, these findings highlight that exercise training may have anti-inflammatory therapeutic potential in MS. Furthermore, Schulz et al. (2004) demonstrated that a 30 min endurance test in trained MS patients (immunomodulatory treatment included IFN-β and copaxone) who have undertaken an 8-week aerobic training programme (cycle ergometry at 60% of VO_2_max; 2 times/week) marginally enhanced levels of soluble IL-6 receptor (sIL-6R) in plasma (Schulz et al., 2004).

### Exercise and MS clinical characteristics

In addition to the alterations in inflammatory signature, physical activity is proposed to target multiple clinical manifestations in MS; however the optimal exercise prescription has not yet been established for MS patients. A body of data illustrates that physical activity is an effective strategy for overall health, which is accessible to most individuals and is without any intolerable side effects that generally coexist with pharmaceutical treatment. Indeed, Learmonth et al. (2014) have shown that a 15 min bout of moderate-intensity exercise had no adverse effects on pain or function and thus no negative impact on MS patients (Learmonth et al., 2014). Similarly, Collett et al. (2011) showed that low, high and combined intensity exercise on a cycle ergometer were all safe protocols for MS patients. Higher intensities may be less tolerated but are postulated to produce faster and larger improvements (Collett et al., 2011). There is sufficient evidence to suggest that exercise among MS patients may have positive effects on aerobic fitness (Briken et al., 2014; Schmidt and Wonneberger, 2014; Skjerbæk et al., 2014), mobility (Kileff and Ashburn, 2005; Rampello et al., 2007; Collett et al., 2011), muscle strength (Golzari et al., 2010), mood/depression (Ahmadi et al., 2013; Briken et al., 2014), fatigue (Ahmadi et al., 2013; Learmonth et al., 2014; Schmidt and Wonneberger, 2014), and cognitive disturbances (Sangelaji et al., 2015; outlined below).

Mobility is a critical concern for individuals with MS, interfering with performance in daily living activities. A high prevalence rate of falls also occurs among MS patients (Finlayson et al., 2006), and a fear of falling can consequently limit mobility in patients. The primary implicating factor of falling among MS patients is due to gait imbalance and muscle weakness, and hence this coincides with restricted activity due to concerns about falling (Matsuda et al., 2012); this causes a vicious cycle and progressive mobility decline, resulting in reduced independence and quality of life. A randomized controlled trial involving supervised balance rehabilitation sessions (10–12 sessions over 3 week) has been shown to improve balance and sequentially significantly reduce fall risk in middle aged MS patients (Cattaneo et al., 2007). Rampello et al. (2007) identified that following an 8 week aerobic cycling program involving 3 × 30 min sessions per week was sufficient to exhibit a significant improvement in overall mobility in MS patients (Rampello et al., 2007). Furthermore, combined resistance-endurance training (24 sessions during 8 weeks) significantly improved muscle strength and balance in MS patients (Golzari et al., 2010). Additionally, the incidence of osteoporosis and fractures are elevated among MS patients due to decreased mobility and the use of steroid medications. Resistance training improves bone strength and muscle power (Gupta et al., 2014) and may be a useful intervention for MS patients to improve overall mobility.

Briken et al. (2014) recently showed significant improvements in aerobic fitness in moderately disabled MS patients following an exercise training programme (arm ergometry, rowing and bicycle ergometry) consisting of 2–3 sessions per week for 8–10 weeks (Briken et al., 2014). Skjerbæk et al. (2014) also recently demonstrated that even severely disabled patients can improve fitness levels in just 10 exercise sessions over 4 weeks through upper body endurance training (Skjerbæk et al., 2014).

Fatigue, a debilitating symptom experienced by most MS patients, further disrupts aspects of everyday activities and interferes with normal life. While the effect of exercise on fatigue in MS patients remains unclear, improvement in fatigue appears to depend on the type of exercise. In one study, analysis suggested that a decrease in fatigue (as determined using visual analog scales) emerged in MS patients after a single bout (15 min) of aerobic cycling (Learmonth et al., 2014). Additionally, Schmidt and Wonneberger (2014) demonstrated that long-term endurance training (3 × 30 min sessions per week for 12 months) significantly improved fatigue (as determined using the Fatigue Severity Scale) in MS patients who had fatigue at baseline (Schmidt and Wonneberger, 2014).

Anxiety and depression are elevated amongst MS patients in comparison to healthy individuals (McCabe, 2005). Data from Ahmadi et al. (2013) showed that both treadmill training and yoga practice (24 sessions over 3 weeks) promote significant improvements in depression and anxiety in MS patients with mild—moderate disability (EDSS: 1–4) as assessed by the Beck Depression Inventory and Beck Anxiety Inventory, respectively (Ahmadi et al., 2013). Additionally, arm and bicycle ergometry training programmes (2–3 sessions/week for 8–10 weeks) promoted a significant decrease in depressive symptoms in moderately disabled MS patients, compared to a control group, as assessed by the Inventory of Depressive Symptoms self-report questionnaire (Briken et al., 2014). Thus, numerous types of exercise exert a favorable effect on depression and anxiety in MS patients, and this is consistent with studies showing beneficial effects of exercise in major depression (Mota-Pereira et al., 2011; Trivedi et al., 2011; Silveira et al., 2013). Importantly, recent studies suggest that the beneficial effect of exercise in depression might be a consequence of counteracting the impact of the associated inflammation on tryptophan metabolism along the kynurenine pathway (Agudelo et al., 2014).

Cognitive disturbances affect ~40–65% of MS patients (Rao et al., 1991) and it is associated with negative consequences on quality of life and further contributes to disability status. Exercise is neuro-protective, improving memory and promoting hippocampal neurogenesis in rodents (Bechara et al., 2013). Intertestingly, hippocampal volume has been shown to increase in response to exercise in both an elderly cohort with probable cognitive impairment (ten Brinke et al., 2014), and cognitively impaired MS patients (Leavitt et al., 2014), and exercise may also enhance cognitive function in MS (Briken et al., 2014; Leavitt et al., 2014). Indeed, Sangelaji et al. (2015) recently demonstrated that combined training (10–20 mins treadmill, 10–20 mins stationary bike and 30 mins balance exercises) of 24 sessions over 8 weeks resulted in a significant increase in long-term storage and permanent long-term retrieval of information, in addition to a significant increase in Digit Symbol Modality Test (DSMT) score among MS patients (Sangelaji et al., 2015).

## Conclusion

MS is a chronic disease accounting for lasting disability among young adults and hence MS rehabilitation is essential for patients to maintain an independent lifestyle and to ensure an improved quality of life. Originally MS patients were advised to avoid exercise since elevated body temperature was suggested to exacerbate symptoms, as observed by Uhthoff (Humm et al., 2004). MS patients demonstrate difficulties with gait imbalance and muscle weakness, which is associated with restricted levels of physical activity. However, since the 1980's, many studies have highlighted that MS patients benefit from exercise. While exercise is essential for good general health and quality of life, it is also helpful to alleviate multiple symptoms associated with MS. A body of literature now suggests that exercise has the potential to modulate MS pathology and may potentially modify the progression of the disease (Dalgas and Stenager, 2012). Indeed, low levels of physical activity correspond with a higher disease burden (increase in T2 lesions and relapse rates) in pediatric MS patients (Grover et al., 2015), suggesting a protective effect of exercise in MS.

There is no cure for MS, with a number of approved medications in the clinic demonstrating proclivity to reduce frequency of relapses and long-term accrual of disability. The observation that disability often continues to worsen despite immunotherapy has prompted some MS patients to seek alternative treatments for the disease. Since MS is an immune-mediated disease and MS patients demonstrate a shift toward a pro-inflammatory signature, neuro-immune signaling represents a clear therapeutic target. Recent data suggests the potential role of the innate immune system in the initiation and progression of MS, and also indicates that exercise may modulate the innate immune system by directly targeting TLR signaling events. Given these findings, characterizing the impact of aerobic exercise on the expression profile of TLRs and associated inflammatory cytokines linked with MS neuropathology requires full investigation. Such investigation will elucidate the clear biological basis for exercise in MS, and will furthermore assist in delineating the therapeutic potential of exercise training in individuals afflicted by the disease.

## Author contributions

ED and KO funded the project. ED and AB wrote the manuscript. OC, AR, BS, SY, OO, AA, GC, and KO critiqued and edited the manuscript drafts.

### Conflict of interest statement

The authors declare that the research was conducted in the absence of any commercial or financial relationships that could be construed as a potential conflict of interest.
